# A TALE-inspired computational screen for proteins that contain approximate tandem repeats

**DOI:** 10.1371/journal.pone.0179173

**Published:** 2017-06-15

**Authors:** Malgorzata Perycz, Joanna Krwawicz, Matthias Bochtler

**Affiliations:** 1 Polish Academy of Sciences, Institute of Biochemistry and Biophysics, Warsaw, Poland; 2 International Institute of Molecular and Cell Biology in Warsaw, Poland; Weizmann Institute of Science, ISRAEL

## Abstract

TAL (transcription activator-like) effectors (TALEs) are bacterial proteins that are secreted from bacteria to plant cells to act as transcriptional activators. TALEs and related proteins (RipTALs, BurrH, MOrTL1 and MOrTL2) contain approximate tandem repeats that differ in conserved positions that define specificity. Using PERL, we screened ~47 million protein sequences for TALE-like architecture characterized by approximate tandem repeats (between 30 and 43 amino acids in length) and sequence variability in conserved positions, without requiring sequence similarity to TALEs. Candidate proteins were scored according to their propensity for nuclear localization, secondary structure, repeat sequence complexity, as well as covariation and predicted structural proximity of variable residues. Biological context was tentatively inferred from co-occurrence of other domains and interactome predictions. Approximate repeats with TALE-like features that merit experimental characterization were found in a protein of chestnut blight fungus, a eukaryotic plant pathogen.

## Introduction

TALEs (transcription activator-like effectors) were first identified in *Xanthomonas* bacteria, which use the proteins as tools to manipulate host gene expression in favor of infection [1]. TALEs are delivered to plant cells via bacterial type III secretion systems [2]. They possess nuclear localization signals that target the proteins to the nuclei of plant hosts of the TALE producer organisms. TALEs bind plant promoter sequences in a sequence specific manner [3, 4]. Specificities are mediated by approximate repeats that consist of two-helix bundles. In the presence of target DNA, the helix bundles are tightly packed against each other so that they can form a right-handed super-helix that wraps around the DNA, matching its rise. TALE repeats differ in two positions in the linker region between helices [3, 4]. The variable residues (termed repeat-variable diresidues or RVDs) co-vary. The second RVD residue, RVD2, directly contacts the nucleobase of the DNA that runs “parallel” (5’-3’ direction along N- to C-direction) to sequence of TALE repeats [5, 6]. Its interaction with the DNA base, by van der Waals interactions and hydrogen bonds, explains the simple cipher that relates the sequence of RVDs in repeats to target DNA sequence (HD → C, NG → T, NI → A, NN→ R, NK→G, HG →Y, NS → M>>K, IUPAC nomenclature for bases). The base at the 5’-end of the recognition sequence is always T, identified by interactions with degenerate repeats upstream of the canonical ones [5, 7].

Non-canonical TALE-like proteins (referred to as TALE-likes) have also been described in prokaryotic organisms other than *Xanthomonas*. These are RipTALs from *Ralstonia solanacearum* [8, 9], BATs (BurrH) from *Burkholderia rhizoxinica* [10–12], and MOrTL1 and MOrTL2, identified in a metagenomic screen of marine bacteria [13–15]. They share common characteristics of tandem repeats and of a (mostly) conserved code. TALEs and RipTALs possess type III secretion signals, but BurrH does not, and so its ability to act as a eukaryotic transcription factor is unclear [12] (deposited coding sequences (CDSs) of both MOrTLs 1 and 2 are partial so we cannot determine any additional features that the full-length MOrTLs could have). Pairwise comparisons of consensus tandem repeat sequences showed that RipTAL repeats were most similar and MOrTL1 and MOrTL2 repeats least similar to a canonical TALE repeat [15].

TALE-like proteins have so far been identified by comparisons of sequences or sequence profiles. We systematically searched UniRef100 for sequences containing 30–43 residue long imperfect tandem repeats that would concentrate sequence variability in a few places in the repeat (like RVDs in TALEs), and then used data mining as well as bioinformatics predictions of protein localization and structure to single out candidates that could potentially function in a TALE like manner. Our screen turned up many known repeats [16], such as ankyrin, agglutinin-like, tetratricopeptide (TPR), EGF-like, and armadillo repeats, which are unlikely to function in a TALE like manner. However, the screen also identified yet undescribed tandem repeats in fungal pathogens parasitizing higher plants. The repeats in a protein of chestnut blight are distantly similar to those in TALEs, with variable residues that could be suitable for DNA interactions.

## Materials and methods

### Database

UniRef100 database was used [http://www.uniprot.org/uniref/], November 2014 version [17].

### RepeatReaper.pl—A program for retrieval of sequences containing 30–43 residue long tandem repeats

**Fig 1:**
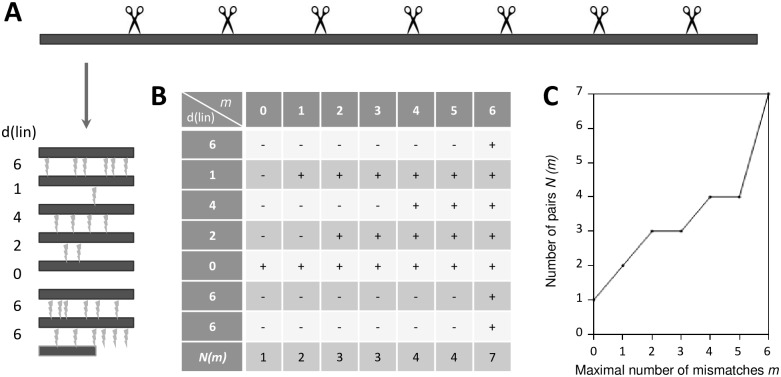
RR operating principle. (A) In order to check for repeats of a given length, RR breaks down a protein sequence into a series of adjacent fragments (shown here as black rectangles), and calculates the number of mismatches (lightning symbols). We refer to this number also as the linear or raw distance to distinguish it from the register-corrected or cyclic distance. (B) RR counts the number *N* of pairs of adjacent fragments that (without any alignment) differ in at most *m* positions. The table shows exemplary analysis for the protein fragments depicted in (A). (C) The *N(m)* fingerprint of a given protein contains information about the repeat content of the protein. The graph shows relation between the number of mismatches (*m*) and the number of fragment pairs that differ in *m* or less positions (*N(m)*), for the protein fragments depicted in (A).

RepeatReaper.pl (RR) is a Perl script (provided as S1 Material) for identification of highly similar adjacent repeats in proteins. RR operates on multi-FASTA files of protein sequences and searches for repeats of user-defined length (here, 30–43) (Fig 1 and S1A Fig). RR divides the amino acid sequence into fragments of the queried length. The program then calculates the number of mismatches (the linear distance, d(lin)) between pairs of adjacent fragments (mismatches are highlighted by lightning symbols in Fig 1A). Next, the program counts the pairs of adjacent fragments that are similar according to its settings. Scoring of fragments as similar depends on the parameter *m* that specifies the maximum acceptable number of mismatches between fragments. A fragment pair is included (illustrated by a “+” sign in Fig 1B) in the *N(m)* count if and only if d(lin) is smaller or equal to *m*. The larger *m*, the lower the stringency of the comparison, and hence the larger the number *N(m)* of similar repeats. The function *N(m)* (illustrated for the example in Fig 1C) can be treated as a fingerprint of a protein sequence that provides information about the repeat content.

Repeat proteins are selected based on selection criteria for *N(m)*. Representative repeats in a protein are selected as the fragments with the smallest combined number of mismatches to all other repeats after register correction. Technically, we define a “cyclic” distance in addition to the “raw” distance between Strings 1 and 2 (S1B Fig). For the calculation of the “cyclic” distance, String2 is cyclically permuted. The number of mismatches is then counted between String1 and all cyclic permutations of String2, and the minimum number is taken as the cyclic distance. A fragment of minimal cyclic distance to all other fragments is chosen as the representative repeat (S1C Fig). The use of cyclic (rather than raw) distances makes the algorithm robust against register shifts due to occasional shorter or longer repeats, as frequently observed in TALE proteins. Some repeats may be built from shorter repeats, either completely (e.g. GAAREGAAREGAARE) or partly (e.g. GAAREGAAREGAARESS). We refer to such repeats as “composite” repeats. In order to automatically identify such repeats, representative repeats are compared with all cyclic permutations except for the identity permutation. For every permutation, the number of mismatches to the non-permuted sequence is calculated. For non-composite repeats, this number is consistently large. For composite repeats, at least one cyclic permutation essentially aligns the original and permuted repeats, leading to a small number of mismatches. The minimum number of mismatches (excluding the identity permutation) is therefore a good indicator for the presence of internal repeats. We classified a repeat as composite when the minimum was smaller or equal to half the repeat length (S1D Fig).

### RepeatCluster.pl—A program for clustering (representative) repeats

A greedy clustering algorithm is used to cluster repeats. For each repeat, the sum of cyclic distances to all other repeats is calculated. The repeat with the smallest combined distance to the other repeats is then picked, and all similar repeats are identified (a “tolerance” threshold of at most five non-matching positions is used). The repeat and the related repeats are set aside as a cluster, and the procedure is repeated for the remaining repeats, until all repeats have been grouped.

### AminoModuleMatch—A program for identification of repeats of set length within a sequence

AminoModuleMatch (AMM) is a Java script (S2 Material) that operates on a FASTA file of a single protein sequence, detecting repeat modules of a set length. Briefly, the script scans protein sequences with a series of queries derived from the investigated protein sequences themselves. Query length and similarity threshold are adjustable. For this analysis, query length was in the range from 30 to 43 and the threshold was set to 80%. The results of the analysis were written into a text file and contained the name and length of the sequence, the number of residues within a repeat module, the numbers and positions of repeats within a protein sequence, and the alignments of all identified module repeats to the query annotated by the fraction of identical residues. The program was used for validation of RR results during RR’s development.

### Quantification of repeat complexity by the Shannon formula

Sequence complexity of a repeat was described by Shannon’s entropy score. The score is defined as the negative of a sum of the products of amino acid frequencies in a typical repeat sequence (*p*_*i*_) and binary logarithms of those frequencies (log_2_(*p*_*i*_)).

$$S=-\sum_{j=1}^{s}p_{i}{\text{log}}_{2}p_{i}$$

### Globularity, secondary structure predictions and PDB scan

Globularity was predicted using GlobPlot [18]. The program identifies regions of globularity (order) and disorder within protein sequences. Its approach is based on a running sum of the propensity for amino acids to be in an ordered or disordered state. In order to avoid “edge” effects, a GlobPlot score was calculated for a concatemer of six tandem repeats (using the default parameters for the batch version). Secondary structure predictions were made using PSIPRED [19]. PSIPRED identifies homologous sequences and predicts secondary structure based on the position specific scoring matrices generated by PSI-BLAST [20]. The Protein Data Bank (PDB) was searched for candidate repeat regions using PDB-BLAST [http://blast.ncbi.nlm.nih.gov/Blast.cgi] and RCSB PDB (http://www.rcsb.org/pdb/home/home.do#Subcategory-search_sequences]) [21]. For the NCBI BLAST PDB search we used the quadruple of repeat motifs.

### Sequence logos of the alignments of the tandem repeats

For a graphic representation of an amino acid multiple repeat sequence alignments, we used sequence logos (SeqLogos) [22] generated using WebLogo online tool [23].

### Variable residues in the repeats

The positions and frequencies of variable amino acid residues (VRs) within tandem repeats were identified using RR by calculating frequencies of individual residues in the columns of repeat alignments. Sequences in which VRs were adjacent, or spaced every 2, 3 or 4 residues (suggesting proximity depending on secondary structure context) were identified manually based on sequence logos.

### Covariation of variable residues

Mutual information (MI) was used to quantify covariation between suitably spaced variable residues (adjacent residues in unstructured regions, or {n, n+2} residues in sheet regions, or {n, n+1}, {n, n+3} and {n, n+4} residues in helical regions), according to the formula:

$$MI\;\left(X;Y\right)=\;\sum_{x\in X}\sum_{y\in Y}p\left(x,y\right){\text{log}}_{2}\left(\frac{p\left(x,y\right)}{p\left(x\right)p\left(y\right)}\right)$$

Diresidue frequencies were illustrated as 2D heat maps.

### Sequence similarity between newly identified tandem repeats and TALE tandem repeat

The Jensen-Shannon divergence (JSD) was used to quantify dissimilarity between pairs of alignments (each alignment is for a set of related repeats) [24]. For residue frequencies *p* and *q* of a given amino acid (*a*) in a given position (*l*), JSD is defined as:

$$\text{JSD}=\frac{1}{2}\sum_{a\in A}p_{l,a}{\text{log}}_{2}\frac{p_{l,a}}{m_{l,a}}+\frac{1}{2}\sum_{a\in A}q_{l,a}{\text{log}}_{2}\frac{q_{l,a}}{m_{l,a}}$$

Where $m_{l,a}=\frac{p_{l,a}+q_{l,a}}{2}$ and A is a set of 20 amino acids.

Alignments were generated using Multiple Sequence Alignment (Muscle) (http://www.ebi.ac.uk/Tools/msa/muscle/) [25]. Frequencies of amino acids *a* in positions *l* were counted using frequency statistics calculator (http://www.csgnetwork.com/documentanalystcalc.html).

### Nuclear localization prediction

We used NucPred [26] (http://www.sbc.su.se/~maccallr/nucpred/cgi-bin/batch.cgi) to predict possible nuclear localization of the proteins. The program assigns a score between 0 and 1 to a protein to describe the probability of it being nuclear (0 predicts no nuclear localization and 1 predicts exclusively nuclear localization). We also used cNLS mapper to search for known nuclear localization sequences within analyzed proteins [27], http://nls-mapper.iab.keio.ac.jp/cgi-bin/NLS_Mapper_form.cgi.

### Prediction of secretion signals

The presence of secretion signals in prokaryotic proteins was predicted using the Effective [28], (http://www.effectors.org/) and SecretomeP [29, 30] (http://www.cbs.dtu.dk/services/SecretomeP/) programs.

### Prediction of transactivation domains

The candidate sequences were checked for presence of putative transactivation domains using the “Nine Amino Acids Transactivation Domain” 9aaTAD Prediction Tool http://www.med.muni.cz/9aaTAD. Three patterns of various stringencies were used: [MDENQSTYG]{KRHCGP}[ILVFWM]{KRHCGP}{CGP}{CGP}[ILVFWM] {CGP}{CGP} which is moderate and described as generally best for mammals and yeast transcription factors; [MDENQSTYG]{KRHCGP}[ILVFWM]{KRHCGP}{CGP}{KRHCGP}[ILVFWM][ILVFWMAY]{KRHC}, which is most stringent and suggested for most yeast TFs, and [MDENQSTYCPGA]X[ILVFWMAY] {KRHCGP}{CGP}{CGP}[ILVFWMAY]XX, which is least stringent and described as best for viral, artificial and other TFs or short transactivation domains w/o suitable hits with stringent patterns.

### Characterization of candidate proteins

NCBI protein-protein blast and protein-nucleotide blast on non-redundant databases http://blast.ncbi.nlm.nih.gov/Blast.cgi were used to search for related repeats, using a quadruple repeat module of the candidate protein as the query. Protein domains were searched using the Pfam online tool http://pfam.xfam.org/search/sequence and the NCBI conserved domain search http://www.ncbi.nlm.nih.gov/Structure/cdd/wrpsb.cgi. STRING analysis was performed to recognize known and predicted protein-protein interactions of selected candidates [31].

## Results

### Search procedure

**Fig 2:**
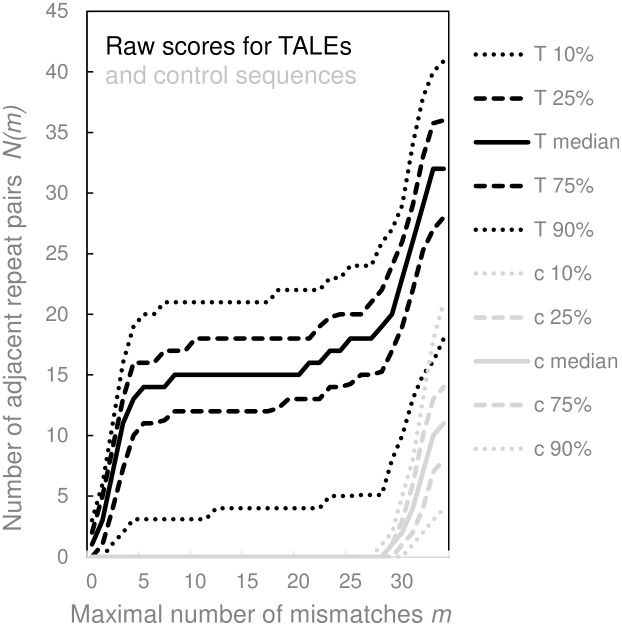
Identification of tandem repeats as a function of number of mismatches allowed. Distribution of tandem repeat scores for a set of TALEs and a random subset of tested proteins from the UniRef100 database. The fingerprint function *N(m)* was calculated for 117376 randomly chosen UniProt100 sequences (grey lines), and all but atypical TALEs (149 out of 182) (black lines). For every allowed number of mismatches *m*, the medians and 10%, 25%, 70% and 80% percentiles of *N(m)* are plotted. The *N(m)* fingerprints of “typical” proteins and TALE proteins have very different shapes.

For this study we designed RR (S1 Material), a program for the high-throughput search of approximate repeats in proteins. RR requires as input a multi-fasta file and calculates a “fingerprint” *N(m)* of a protein sequence that is informative about the repeat content (Fig 1, S1 Fig). The parameter *m* defines the stringency of the comparison between fragments (and hence corresponds to their similarity). Technically, *m* is defined as the maximum allowed number of mismatches between adjacent fragments required for scoring them as repeats. For *m = 0*, only identical fragments are counted as repeats. The larger *m*, the more mismatches are accepted. Typical proteins have dissimilar fragments that do not contribute to *N(m)* when comparisons are made with high or medium stringency (low or medium *m* values, Fig 2, grey lines). Only when comparison criteria are very relaxed (*m* values approaching the fragment length), the fragments are scored as repeats (Fig 2, grey lines). TALE proteins have very different *N(m)* fingerprints than “typical” proteins (Fig 2, black lines). *N(m)* is small only when *m* is so small that adjacent repeats are scored as dissimilar. A slight relaxation of the similarity criteria (small *m*) suffices for the number of adjacent repeats to rise sharply. As *m* increases further, *N(m)* has a plateau, which reflects the lack of similarity of fragments outside the repeat region. Only for large *m* approaching the repeat length, *N(m)* fingerprints for TALEs and “typical” proteins become similar (Fig 2, black lines).

149 of the 182 TALE control proteins (82%) had at least 10 adjacent repeat pairs differing in no more than 5 positions. The others either contained few TALE repeats altogether (some sequences were explicitly annotated as incomplete) or contained many 33-amino acid repeats interspersed between the more typical 34-amino acid repeats. For all 149 TALE proteins with at least 10 “similar” adjacent repeats, less than half of the adjacent repeats were identical. We reasoned that a recovery rate of 82% should be sufficient to identify at least some members of a putative novel TALE-like protein family, and did not relax criteria further to avoid identifying too many likely “false positives” that required manual checking.

**Fig 3:**
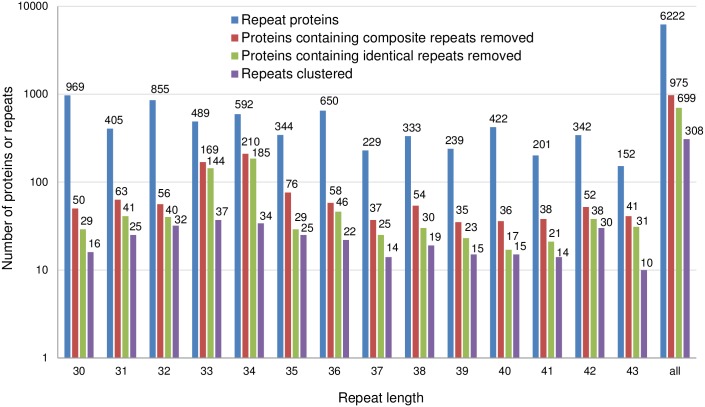
Numbers of tandem repeat sequences identified in the search. Number of protein sequences containing at least 10 repeat pairs (11 similar adjacent fragments) of 30–43 aa residues (blue), and number of remaining sequences after excluding composite (red) and then identical repeats (green). Purple bars show the number of repeat families after clustering.

Next, we searched the entire UniRef100 dataset of altogether ~47 million protein sequences for repetitive sequences, applying the criteria met by most of the TALE proteins (minimally 10 repeat pairs differing in at most 5 positions). Possible repeat lengths in the range from 30 to 43 were tested. Approximately 6000 candidate sequences were identified (for all repeat lengths combined) (Fig 3).

A breakdown of candidate sequences (or representative repeats) according to repeat length indicated major variation (note that the logarithmic scale of Fig 3 partly obscures this effect). In general, the shorter the repeat, the more proteins were found. When the queried repeat length was a prime number, fewer proteins were identified than otherwise (two-sided Kolmogorov Smirnov test, p = 0.015). The latter observation suggested that many repeats were “composite” or built from shorter repeats (e.g. three consecutive repeats of length 10 form a repeat of length 30). The presence of composite repeats at this stage of analysis was confirmed by manual inspection using another tool of our design, AMM (S2 Material). In the next step we used RR to automatically identify and exclude composite repeats from the set. RR identified composite repeats by comparing repeat sequences with all cyclic permutations (excluding identity), and classified repeats as composite when at least one permutation was identical in more than half of all positions (S1D Fig). Exclusion of composite repeats brought the number of candidate protein sequences down to ~1000 (Fig 3).

In many tested cases, more than half of the adjacent repeats were identical, suggesting that the repeats in these proteins did not have the necessary variability required for a “cipher” and did not merit further consideration. Altogether, the pre-screen thus identified ~700 protein sequences for more detailed further analysis. In order to cluster proteins according to their representative repeat, we wrote the Perl script RepeatCluster.pl that implements a register-independent greedy clustering algorithm. Clustering bundles the ~700 repeats into ~300 clusters. By far the largest cluster identified in this manner is formed by the TALE proteins (146 members). The second largest cluster is already much smaller (47 members, from prepilin sequences), and most clusters contain repeats from only a single amino acid sequence.

### Filtering based on complexity of a repeat (Shannon score)

**Fig 4:**
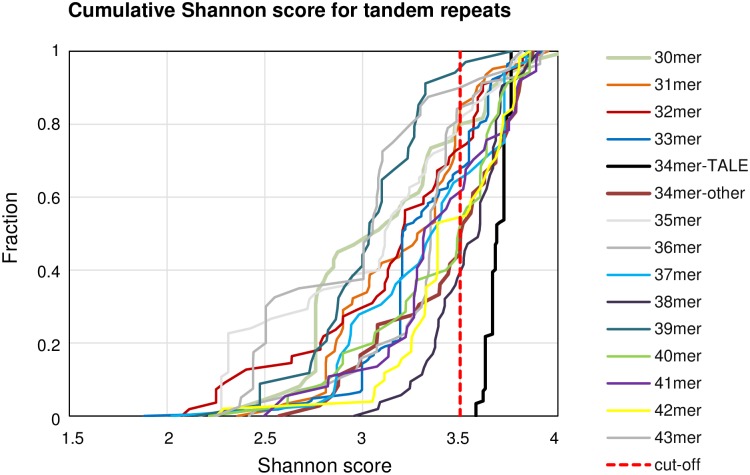
Cumulative Shannon scores for tandem repeats. Cumulative Shannon complexity of the representative tandem repeats from the TALEs identified in the search (black line) and all the other (non-TALE) hits (colored lines, clustered by repeat length). The dotted red line marks the minimal complexity of repeats considered further.

We used Shannon score as a measure of complexity of a repeat sequence. For an alphabet of 20 amino acids, the Shannon scores range from 0 (just one type of aa in the repeat, frequency = 1, log_2_1 = 0) to 4.32 (all amino acids equally represented, aa frequencies = 0.05, log_2_0.05 = −4.32). Shannon scores for the 699 sequences derived in the previous steps (Fig 4) fell between 1.88 and 4.05. The scores for TALE repeats were in the narrow range from 3.58 to 3.76, reflecting the similarity between repeats. 85 repeat types (including TALEs), representing 320 individual sequences had Shannon scores similar to or higher than TALE repeats, and were analyzed further (S3 Material). All 146 TALEs passed this step.

### Taxonomy of identified sequences

**Fig 5:**
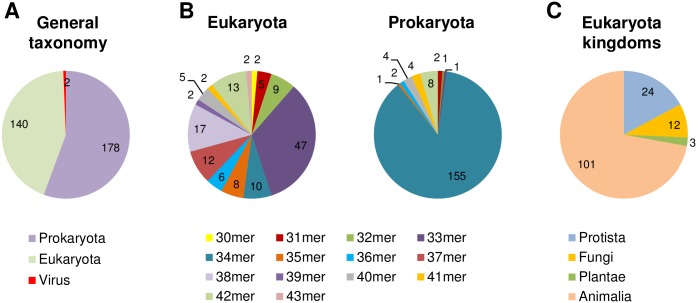
Taxonomy of the hits. (A) Participation of all identified filtered hits in taxonomic domains. (B), Participation of individual classes of hits in taxonomic domains. (C) Participation of sequences of different kingdoms within Eukaryota.

TAL effectors are prokaryotic proteins of a bacterial plant pathogen *Xanthomonas*. Looking for possible similarities on this level, we checked the taxonomy of candidate proteins (Fig 5). Out of 320 sequences in our screen, 178 were prokaryotic (including 147 TALEs), 140 were eukaryotic, and 2 were viral. Among the eukaryotic proteins, a substantial subset was derived from animals (101), then protists (24), fungi (12) and plants (3).

### Nuclear localization predictions for the candidate proteins

**Fig 6:**
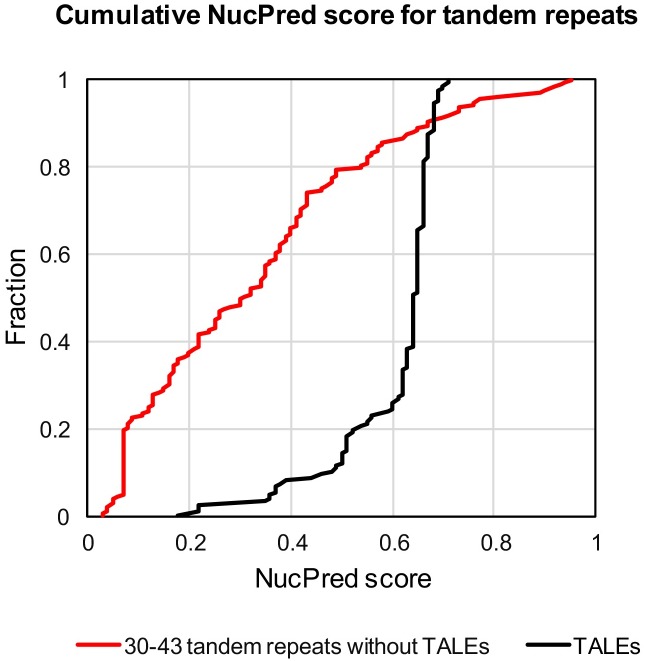
Cellular localization prediction. Cellular localization of the proteins was predicted using NucPred. The cumulative scores for protein sequences containing tandem repeats (red) and TALEs (black) are shown. Scale: 0 means no nuclear localization, 1 means exclusively nuclear localization.

We used NucPred (http://www.sbc.su.se/~maccallr/nucpred/ [26], to predict if a protein spends at least some time in the nucleus. NucPred score falls between 0 and 1, and the higher the score, the higher probability that a given protein can localize to nucleus. The control set of *Xanthomonas* TALE and Avr proteins, which are known to localize to nucleus, in this screen had a mean score of 0.60, ranging from 0.18 to 0.71. 75% of all TALEs had NucPred score between 0.6 and 0.71, and 90% had a NucPred score between 0.46 and 0.71 (Fig 6). Analysis of a control TALE set with cNLS mapper (set for a cut-off of 4 and search for both single and bipartite NLS) identified NLS in 94% (130/138) of the full-length sequences (and in 89% of all the TALE sequences identified in this search, including partial). For all the other sequences (TALEs excluded), the lowest NucPred score was 0.03 (highly unlikely to be nuclear), and the highest score was 0.95 (very likely to be nuclear). For 65% of the sequences, the NucPred scores were equal of higher than the minimum score for the control TALE set (0.18), and 25% had NucPred score equal to or higher than 0.46 (which is true for 90% of the TALEs). Analysis with cNLS mapper was done for the best candidates (summarized in Table 1) and identified NLS in 50% (7/14) of those sequences. As we observed a spread in NucPred scores in TALEs themselves, we used this score only descriptively and not for filtering.

**Table 1:** Novel tandem repeats identified in the search.

Repeat name	Repeat sequence	Repeat length	UniRef100 protein ID	Protein name	Organism	Kingdom	Shannon score of the repeat	Significant PDB hits of a quadruple repeat seq	Highest taxonomic node of the homologs	Number of BLAST nr-seq hits	Additional domains	TAD	NucPred score	cNLS mapper
#32	VKITAEVVQAAAGNWRSGREVMALLLDQRSDE	32	W1I7I9	Pseudo vic protein	*Cryphonectria parasitica*	*Fungi*	3.5900	None	*Leotimyceta*	>100	HET (not in the repeat region); MPN	Yes	0.35	No
#37	VSAGMARLGGCVRTAGNRGTGATIASRQEGDMEAFPR	37	G5AAP8	Putative uncharacterized protein	*Phytophthora sojae*	*Fungi*	3.600	None	*Phytophtora*	4	None	Yes	0.25	No
#38	ISMVPADVREAGMSKSHVVTSRAASPRCAKCLDQSMSR	38	C9ZJS6	Putative uncharacterized protein	*Trypanosoma brucei gambiense*	*Protista*	3.6685	None	cellular organisms	38 (22 e<0.05)	None	Yes	0.54	No
			C9ZJS7	Putative uncharacterized protein							Deacetyl_PgaB (partial)	Yes	0.73	No
			Q586F1	Uncharacterized protein							Deacetyl_PgaB (partial)	Yes	0.77	No
			Q586F2	Uncharacterized protein							None	Yes	0.54	No
TALE	LTPDQVVAIASNGGGKQALETVQRLLPVLCQDHG	34 (33–35)	numerous	numerous	*Xanthomonas*	*Bacteria*	3.7238	TALE repeat	*Xanthomonas*	>100	Acidic AD	Yes	0.6 (0.18–0.71)	Yes

### Filtering based on secondary structure prediction of the tandem repeat regions

**Fig 7:**
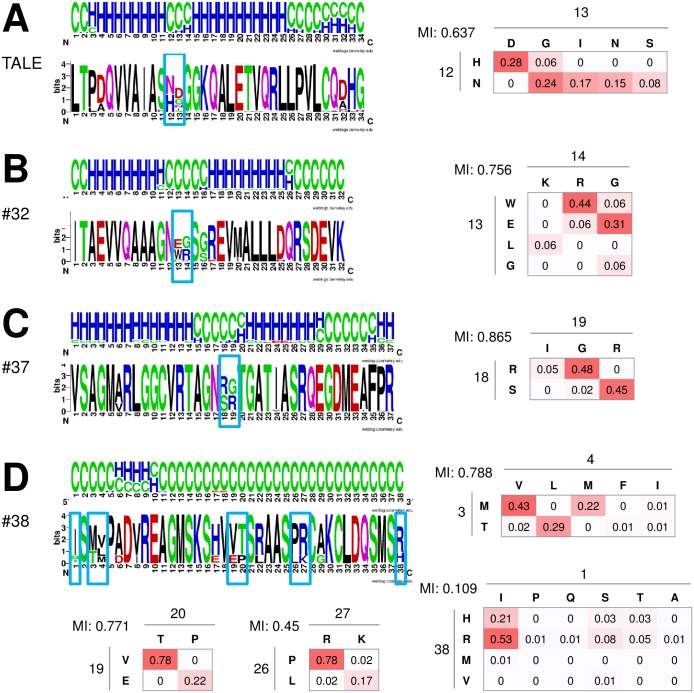
Locations, identities, and covariation of variable residues. Sequence logos for the repeats of (A) TALE; (B), #32; (C), #37; (D), #38 with secondary structure annotation (expressed as frequency plots). All residues in a repeat are numbered and the variable residues are boxed. Heat maps show frequencies of particular pairs of variable residues in the given positions, calculated for the total number of repeats, which was (A) 2733; (B) 16; (C) 44; and (D) 105. Covariation of variables is also described by mutual information (MI).

The TAL effectors display a characteristic secondary structure of their tandem repeats compatible with DNA interactions. Each repeat consists of 2 helices that are linked by a short loop, within which the variable diresidues are located. In the analyzed sequences of candidate proteins, we looked for patterns that may be consistent with a putative interaction with DNA. First, repeat globularity was scored using the standalone version of GlobPlot http://globplot.embl.de [18], using 6x the repeat sequence to avoid “edge” effects. Sequences that had a score 0.5 or higher (meaning at least half of the sequence was predicted to be ordered / globular) were analyzed further with PSIPRED [19], where the full repeat regions were submitted. The secondary structure predictions for all the repeats of a given protein were aligned to make a consensus structure prediction for a repeat. The averaged prediction for a single repeat was presented as a frequency plot produced with WebLogo, and compared against the sequence logo of the given repeat, to map the structural context of the variable residues (a selection of those comparisons is presented in Fig 7). We were looking for pairs of spatially adjacent variable residues. Repeats not predicted to contain helices or strands, repeats with only one variable residue, or repeats with variable residues not predicted to be adjacent according to secondary structure were not considered further. Not counting the TALE repeats, 42 repeat types representing 84 protein sequences passed this step.

### Filtering out the known non-TALE tandem repeats

The identified repeats (other than TALEs) represented not only novel types (summarized in Table 1), but also some of the already described types; we identified them with UniProt, Pfam, PDB and CD-searches. We found the following types of tandem repeats within our set: armadillo, ankyrin, HEAT, WD-40, tetratricopeptide (TPR), pentatricopeptide, agglutinin, and agglutinin-like. For further analysis we focused on the identified tandem repeats that did not show similarity to any motifs deposited in PDB so far. After this filtering step we had 5 repeat types represented by 14 sequences (2 of the logos came from sequences that have been since withdrawn from the newest version of UniRef100). The remaining 3 types of novel repeats (represented by 6 sequences) are shown in Table 1 and Fig 7. In the following, the repeats are designated according to repeat length.

### Residue variability analysis

We selected candidate sequences with variable residues located either in a loop region (as in TALEs) or in α-helices and β-strands with a separation suggesting spatial proximity of side chains (every other residue for β-strands, residues separated by two or three amino acids for α-helices). A cipher like the TALE cipher implies covariation of variable residues. As a measure of covariation, we used mutual information (MI). For the independent variables, or for non-variable pairs, MI = 0. The higher the MI, the better one variable residue can be predicted given knowledge of the other (MI also increases with the increase of variability). The calculated MI for TALE repeats variable diresidues is 0.637 (based on: frequencies of first variable (x) N 0.650, H 0.348; frequencies of second variable (y): G 0.305, D 0.285, I 0.169, N 0.147, S 0.080; frequencies of pairs (x,y): HD 0.279, NG 0.243, NI 0.167, NN 0.146, NS 0.079, HG 0.06). Therefore we calculated MIs for the pairs of variables present in the protein sequences from our screen (Fig 7 shows the results for a selection of the sequences with the “best” structural context of variables). The variables are also presented as heat maps showing the frequencies of individual residues in given positions (Fig 7). For a set of TALE proteins, 4–6 pairs of variable residues predominated (Fig 7A). In most candidate repeats, as in TALEs themselves, some pairings of variable residues were more frequent than others. Mutual information was equally high or higher than for TAL effectors in some cases (Fig 7).

### Sequence similarity between tandem repeats identified in this study and TALE tandem repeats

**Fig 8:**
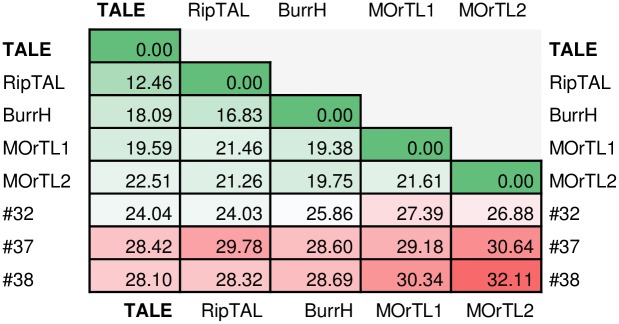
Jensen-Shannon divergence scores for pairwise alignments of tandem repeat arrays. Jensen-Shannon divergence (JSD) of tested tandem repeats. The matrix shows resulting JSD values for the paired alignments of the repeat arrays from the tested proteins: TAL effectors (TALE), TALE likes: RipTAL, BurrH, MOrTL1, MOrTL2, and proteins that contained repeats #32, #37 and #38 (as described in Table 1). Larger JSD values indicate higher divergence.

We wanted to check whether the new tandem repeats found in our search were distinct from the canonical TALE tandem repeats, and tandem repeats of the TALE-likes: RipTAL, BurrH, MOrTL1 and MOrTL2. Therefore we first determined the best-fitting pairwise alignments of representative repeat sequences using MUSCLE. Then we used Jensen-Shannon divergence to compare the repeat logos (i.e. repeat alignments). First, the repeats of each type were aligned (TALE, 17 repeats; RipTAL, 16 repeats; BurrH, 22 repeats; MOrTL1, 7 repeats; MOrTL2, 13 repeats; #32, 16 repeats; #37, 44 repeats; #38, 86 repeats). Then, the alignments were compared against each other and JSD was calculated based on individual amino acid frequencies in corresponding positions of the two logos (shown in Fig 8). For identical sequences the JSD score is 0, and the more they differ, the higher the score. We found that newly identified tandem repeats were less similar to TALEs than TALE-likes described so far.

### Prediction of a presence of the transactivation domains within the candidate sequences

**Fig 9:**
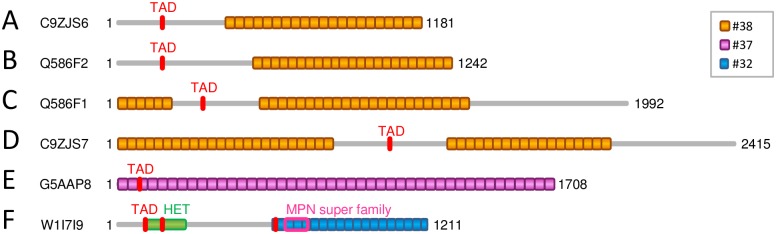
Schematic structure of tandem repeat proteins. Proteins: (A) C9ZJS6, (B) Q586F2, (C) Q586F1 and (D) C9ZJS7 contain repeats of type #38 and TAD; (E) Protein G5AAP8 contains tandem repeats of type #37 and TAD; (F) Protein W1I7I9 contains tandem repeats of type #32, HET domain, MPN domain and triple TAD. Please refer to Table 1 for repeat sequences and descriptions.

This prediction was performed for a set of controls (TALE, BurrH, RipTAL, MOrTL1 and MOrTL2) and the best candidate sequences (Fig 9 and S1 Table). Using the Nine Amino Acids Transactivation Domain 9aaTAD Prediction Tool we were able to identify stringent TAD patterns in the controls: TALE [UniProtKB: AEQ98589.1] and BurrH [UniProtKB: E5AW45], and moderate stringency TAD patterns in partial sequences of MOrTL1 [UniParc: ECG96325.1] and MOrTL2 [UniParc: EBN91409], and also in RipTAL [UniProtKB: Q8XYE3]. Among the candidate proteins, sequences of repeat cluster #38 [UniProtKB: C9ZJS6, C9ZJS7, Q586F1 and Q586F2] contained matches to the most stringent TAD pattern. Less stringent patterns were found in pseudo vic protein [UniProtKB: W1I7I9] containing repeat #32, and putative uncharacterized protein [UniProtKB: G5AAP8] containing repeat #37.

### Other protein domains predicted in the top candidate sequences

To investigate a potential of investigated proteins to be nuclear, in addition to NucPred and 9aa TAD predictions, we analyzed the sequences with conserved domain (CDD-search), and protein domain (Pfam) servers (see Table 1 for a summary and Fig 9 for a schematic). The uncharacterized protein product from *Phytophthora sojae* (strain P6497) (Soybean stem and root rot agent) (*Phytophthora megasperma f*. *sp*. *glycines*) containing repeat #37, did not have identifiable domains. Sequences clustered by repeat #38 either did not contain known domains (C9ZJS6, Q586F2), or contained only a partial poly-beta-1,6-N-acetyl-D-glucosamine N-deacetylase PgaB of *Staphylococcus species / E*. *coli* (identified with CD, not with Pfam; residues 444–511 fragment of 611 domain) (C9ZJS7, Q586F1). In the repeat #32 containing pseudo vic protein W1I7I9, we identified a HET domain, typically associated with heterokaryon incompatibility, a system of self /non-self-recognition in ascomycete fungi that limits genetic exchange [32, 33]. Also, an MPN domain was present (also known as Mov34, PAD-1, JAMM, JAB, MPN+ domain), best known for its occurrence in the 26S proteasome, the signalosome, and elF3 [34] and typically having an isopeptidase activity [35]. The MPN domain in sequence W1I7I9 is closely related to the MPN domain in BRCC36, a subunit of nuclear complex BRCA1-A, which is targeted to DNA damage foci after irradiation, and specifically disassembles K63-linked polyUb [36, 37].

## Discussion

Canonical TALEs have highly similar repeats, with only few variable amino acids, among them the RVDs. Only one of the RVDs (the 13^th^ amino acid) interacts with a target base, whereas the other variable residue has a structural role [5, 6]. Despite the lack of direct contacts of this residue with the target base or its complement, the base contacting and non-contacting residues point in similar directions and show covariation. Here, we report a search for proteins containing highly similar repeats that contain only a few variable residues in conserved positions. Although we are aware that repeats may implement a TALE-like cipher with only a single variable residue, we limited the search to repeats with two co-varying residues, as in TALEs. The identified proteins could in principle interact with DNA in a TALE-like manner, but other explanations for approximate repeats with variation confined to fixed positions in the repeats are also possible. Reassuringly, the identified repeats were usually characteristic not for single proteins, but were shared between larger groups of proteins, suggesting that they were genuinely expressed and not the result of mis-annotation (for example, false conceptual translation of repetitive regions due to erroneously assigned splicing sites). Many families contained variable residues that were predicted to cluster in one spatial region, suggesting that the variable residues may interact with a variable polymer. In the case of TALEs, this is of course double stranded DNA, but in novel cases, it could in principle be single stranded DNA, RNA, or even a completely different polymer (a repetitive structure of the extracellular matrix, a polysaccharide, a glycan chain, etc).

Although this search was designed to identify novel tandem repeats similar to TALEs, it does not exhaust the search for TALE-likes. The selection criteria (at least 10 pairs of adjacent repeats in the protein, and at most 5 differences between the adjacent repeats) were set to emulate the features of TALE proteins. They were relaxed enough to retrieve 82% of the TALE sequences deposited in UniRef100 DB, but strict enough to filter out partial sequences (some TALEs, MOrTL1, identified in a metagenomics study and present in the database as a protein fragment containing 7 tandem repeats). The selection criteria also excluded proteins containing repeats of higher variability (including BurrH and MOrTL2). The identified proteins resembled TALEs with respect to the number and variability of repeats. The sequences of repeats in newly identified proteins were not required to be similar to the sequences of TALE repeats, and indeed, most identified repeats tuned out not to be similar to the TALE repeats (see Fig 8 for examples). At the sequence level, repeats #32 (in the pseudo vic protein W1I7I9 from pathogenic fungus *Cryphonetria parasitica*), #37 (in fungal pathogen *Phytophtora sojae* uncharacterized protein G5AAP8), and #38 (in *Trypanosoma brucei* uncharacterized proteins C9ZJS6, C9ZJS7, Q586F1, Q586F2) lacked not only significant similarity to TALEs, but also to other proteins in the Protein Data Bank (PDB).

Apart from the tandem repeats that interact with DNA, TALEs contain eukaryotic nuclear localization signals (NLS) and acidic activation domain (AD) that are key to their function in infected plants. We used online tools to detect NLS and AD in protein sequences derived from the screen. Although the activation domain in TALEs is roughly 230 amino acids long, we decided to use 9aa TAD detection tool because it represents the smallest known denominator for a broad range of yeast and animal transcription factors [38], and it identifies a 9aa TAD motif within TALE AD (YAFDEAMTQ, at moderately stringent pattern settings).

Three types of tandem repeats fulfilled all the criteria set in this search, namely #32, #37 and #38 (Table 1, Fig 7B–7D). Interestingly, all three repeat types are found in organisms with a parasitic lifestyle. The #38 repeats are found in protozoan proteins, and repeats #32 and #37 occur in fungal proteins. The fungal producers of proteins containing repeats #32 and #37 occupy a similar ecological niche as the TALE producing bacteria parasitizing higher plants. Unfortunately, the proteins containing repeats #32 and #37 are not well characterized and do not have entries in the pathogen host interaction database (PBI-base) [39] that could shed light on their exact biological role.

Repeat #38 (Fig 7D) is present in multiple copies in the trypanosomal proteins C9ZJS6, C9ZJS7, Q586F1, Q586F2 (Fig 9A–9D). The repeat has a Shannon score of 3.67, placement of variable residues in regions of the repeat without clear secondary structure, and a mutual information score high enough to be compatible with a possible cipher. All four trypanosomal proteins possess a predicted TAD, and their NucPred score is 0.54–0.77. However, a NLS was not detected by NLS Mapper. The trypanosomal proteins do not seem to have any other functional conserved domains. Proteins identified as similar by a BLAST search map to diverse domains of life, arguing against a TALE-like role of these proteins. The repeats are at most distantly related or unrelated to the TALE, BurrH, RipTAL, MOrTL1 and MOrTL2 repeats as judged by JSD scores of 28.10, 28.32, 28.69, 30.34 and 32.11, respectively.

Repeat #37 (Fig 7C) occurs in protein G5AAP8 of the fungal plant pathogen *Phytophtora sojae*. The protein has 44 tandem repeats of 37 amino acids (Fig 9E), but unlike TALEs, it does not have other domains. The Shannon score of the repeat is 3.6. Variable residues of the repeat are placed in a favorable structural context (a loop between two helices, as in TALEs), and the mutual information score of 0.87 is high. However, there are only 4 types of variable pairs, in principle just enough to distinguish four bases, compared to six or more RVDs occurring at significant frequencies in TALEs. Despite the prediction of a TAD, the NucPred score of the protein is rather low (0.25) and a NLS was not detected by NLS Mapper. The highest taxonomic node of the proteins similar to G5AAP8 is *Phytophtora*, and there are only four such proteins in the database. The repeat is distinct from the TALE, BurrH, RipTAL, MOrTL1 and MOrTL2 repeats (JSD scores of 28.42, 29.78, 28.60, 29.18 and 30.64, respectively).

Repeat #32 (Fig 7F) is from the pseudo vic protein W1I7I9 of a chestnut blight fungus *Cryphonectria parasitica*, an ascomycete. The repeat has a Shannon score of 3.59, placement of variable residues loop region between two predicted helices, and a mutual information score high enough to be compatible with a possible cipher. W1I7I9 is likely to be secreted according to the fungal secretome knowledge base (FunSec) [40]. The codon adaptation index (CAI) for the W1I7I9 encoding gene (HG799050.1) was more favorable for expression in plant than in fungus (CAI for *C*. *parasitica* = 0.619, CAI for *C*. *molissima* = 0.798). This is reminiscent of TALEs that have CAIs closer to their plant hosts than to their *Xanthomonas* producer organisms. The GC content of HG799050.1 was 50.04%, in between the average GC contents of *C*. *parasitica* (54.89%) and chestnut *Castanea molissima* (46.46%) genes. Together, these findings are consistent with the possibility that W1I7I9 may be a plant protein adapted by fungi and/or a protein encoded in fungi for expression in plants.

W1I7I9 has a low NucPred score and we were not able to detect a NLS using NLS mapper, but the presence of HET and MPN domains and of a TAD motif in the protein may suggest a nuclear role. Jensen-Shannon divergence between the alignment of repeats in W1I7I9 and alignments of TALE and TALE-like proteins shows that the repeat family is an outgroup to TALE and TALE-like proteins (Fig 8). Nevertheless, the similarity to TALEs is higher than for almost all other repeats in this study, including those built from two consecutive helices. Similarly to TALE proteins, the RVDs (EWLG/RGK) are located in a loop region, but differ from those found in TALE and previously characterized related proteins. Most of the residues can act as hydrogen bond donors or acceptors to DNA bases (E, R, K, also W). In TALEs, the “small” glycine (G) and “large” isoleucine (I) are involved in selection of thymine, with maximal space requirements in the outer major groove, and adenine, with minimal space requirements in the outer major groove, respectively. Such shape selection could operate more generally. Also, in TALEs a W residue is involved in the recognition/coordination of the most upstream base of the target sequence [5]. Analysis of co-variation of variable residues in W1I7I9 shows that certain combinations of amino acids are preferred similar to what is seen for TALE repeats. The number of repeats in W1I7I9 is comparable to the number of repeats in a typical TALE protein (15.5–19.5 as stated in [1]), and would thus be sufficient to allow unique addressing of sequences in a plant genome.

In summary, our search of near-identical repeats of similar length to TALE repeats with sequence variation limited to only a few positions has uncovered a number of previously poorly characterized repeats, which may be suitable to bind repetitive polymers. Although this was not a requirement of the search, many of the repeats consisted of two helices connected by a linker often harboring the repeat variable residues, and thus exhibited a TALE like architecture. Intriguing similarity to TALE proteins, with respect to biological context and sequence/structure features, was found for sequence W1I7I9 from chestnut blight fungus. Whether this similarity is biologically meaningful, and whether W1I7I9 indeed binds DNA in a sequence and RVD-dependent manner remains to be checked experimentally.

## Supporting information

S1 Fig Definitions and algorithms. (A) Repeat score of the protein. In order to check for repeats of a given length, RR breaks down a protein sequence into a series of adjacent fragments of this length, and then counts the number of *N* of pairs of adjacent fragments that (without any alignment) differ in at most *m* positions. Comparing adjacent fragments produces so called “raw” distances (linear distance, d(lin)). In the next step, the register of the repeat is determined using cyclic permutation of the most representative repeat in the protein (String1), as shown in panel (B). Cyclic permutation of String1 produces a number of String2, for each of which the calculation of distance (that is, the number of mismatches with an adjacent fragment) is repeated. The resulting permutation which has the lowest number of mismatches with all the other fragments is then taken as the cyclic distance. (C) A fragment with minimal cyclic distance to all other fragments is chosen as the representative repeat. (D) Identifying composite repeats. Cyclic distance measure can be also used for elimination of the repeats that are shorter than requested (like divisors of the requested number). String1 –sequence of the repeat; cyc. perm.–cyclic permutation of the repeat; d(lin)–distance (difference) between the repeat and its permutation. We define composite repeat as a repeat that is shorter than originally set, either because it is a sum of the divisors of the requested length (for example, PREPRE, identified by RR as a repeat of 6, is in fact a double PRE repeat of 3), or because it is shorter by just a few residues so it could still be identified by RR as similar enough to the neighboring fragment in spite of the register shift. In order to exclude composite repeats from the analysis, representative repeats are compared with all cyclic permutations, except for the identity permutation. If the repeat is unique (non-composite), the number of differences between repeats should be similar, regardless of permutations (as shown for VPERVL). For composite repeats, at least one cyclic permutation essentially aligns the original and permuted repeats, leading to a small number of mismatches (as shown for PREPRE). The minimum number of mismatches (excluding the identity permutation) is therefore a good indicator for the presence of internal repeats. We classified a repeat as composite when the minimum was smaller or equal to half the repeat length.
(TIF)

S1 Material RepeatReaper.pl code. (ZIP)

S2 Material AminoModuleMatch code. (ZIP)

S3 Material Protein sequences with Shannon score of the repeats >3.5. This pack contains txt and e.fasta files resulting from RR analysis which included identification of tandem repeat, removal of constitute repeats, removal of identical repeats and removal of repeats of Shannon score <3.5. The files contain protein IDs, consensus repeat sequences, repeat lengths and Shannon scores of the repeats for 320 sequences that constituted 85 tandem repeat clusters.
(TAR)

S1 Table TAD prediction. The table presents transactivation domains identified in protein sequences with Nine Amino Acids Transactivation Domain 9aaTAD Prediction Tool http://www.med.muni.cz/9aaTAD. Stringency patterns re described as: Most stringent: MDENQSTYG] {KRHCGP} [ILVFWM] {KRHCGP}{CGP}{KRHCGP}[ILVFWM][ILVFWMAY]{KRHC}, Moderate: [MDENQSTYG] {KRHCGP} [ILVFWM] {KRHCGP} {CGP} {CGP} [ILVFWM] {CGP} {CGP}, Less stringent: [MDENQSTYCPGA] X[ILVFWMAY] {KRHCGP} {CGP} {CGP} [ILVFWMAY]XX.
(DOCX)

S1 Text STRING analysis results. (DOCX)

## Abbreviations

RVDs: repeat variable diresidues
TAL: transcription activator-like
TALEs: TAL effectors
VRs: variable amino acid residues
