# Supplementary figures and images for: A TALE-inspired computational screen for proteins that contain approximate tandem repeats
Source: PLoS One. 2017 Jun 15;12(6):e0179173. doi: 10.1371/journal.pone.0179173 (PMC5472282; doi:10.1371/journal.pone.0179173)

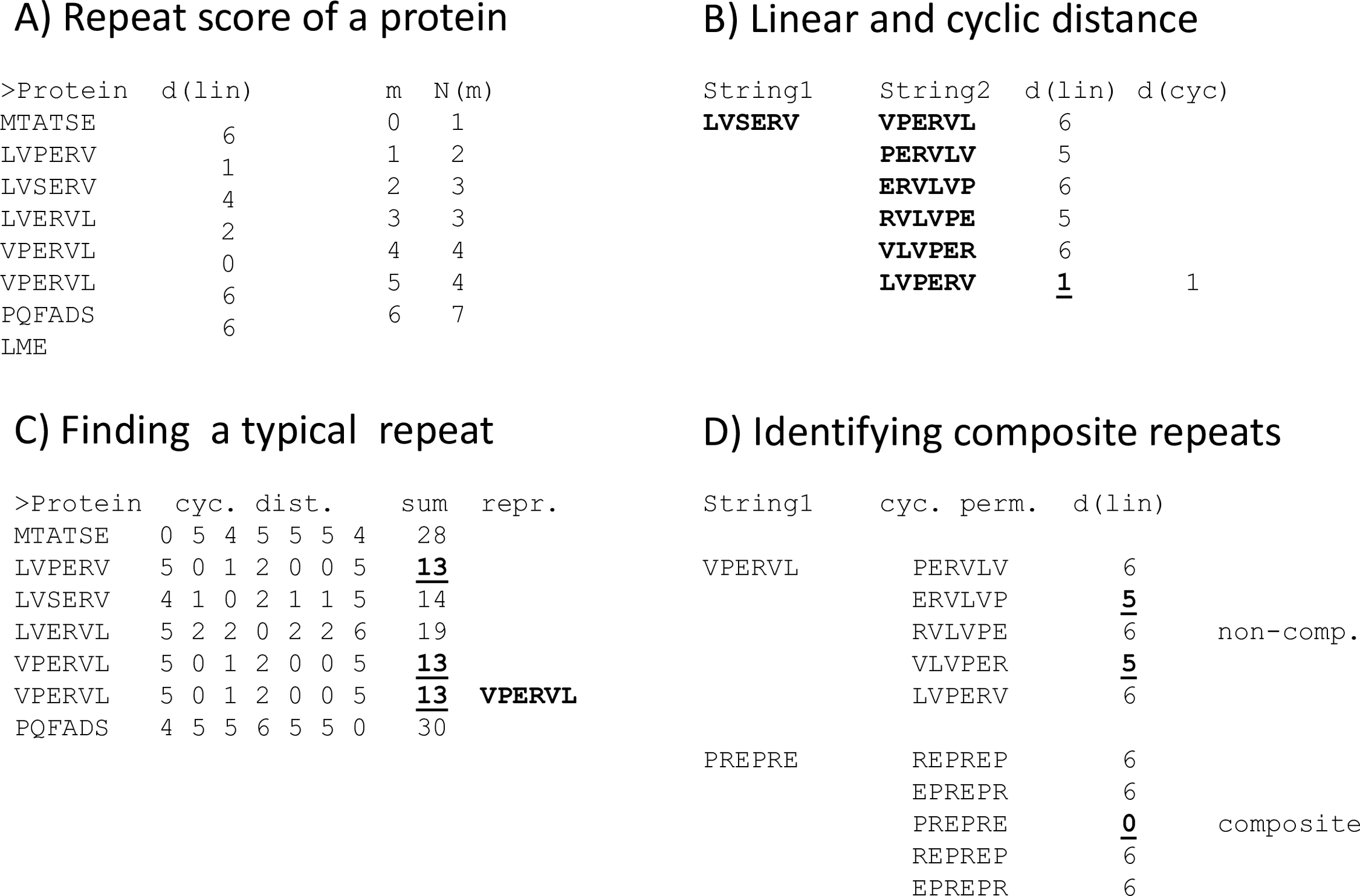

Supplement: S1 Fig — (A) Repeat score of the protein. In order to check for repeats of a given length, RR breaks down a protein sequence into a series of adjacent fragments of this length, and then counts the number of N of pairs of adjacent fragments that (without any alignment) differ in at most m positions. Comparing adjacent fragments produces so called “raw” distances (linear distance, d(lin)). In the next step, the register of the repeat is determined using cyclic permutation of the most representative repeat in the protein (String1), as shown in panel (B). Cyclic permutation of String1 produces a number of String2, for each of which the calculation of distance (that is, the number of mismatches with an adjacent fragment) is repeated. The resulting permutation which has the lowest number of mismatches with all the other fragments is then taken as the cyclic distance. (C) A fragment with minimal cyclic distance to all other fragments is chosen as the representative repeat. (D) Identifying composite repeats. Cyclic distance measure can be also used for elimination of the repeats that are shorter than requested (like divisors of the requested number). String1 –sequence of the repeat; cyc. perm.–cyclic permutation of the repeat; d(lin)–distance (difference) between the repeat and its permutation. We define composite repeat as a repeat that is shorter than originally set, either because it is a sum of the divisors of the requested length (for example, PREPRE, identified by RR as a repeat of 6, is in fact a double PRE repeat of 3), or because it is shorter by just a few residues so it could still be identified by RR as similar enough to the neighboring fragment in spite of the register shift. In order to exclude composite repeats from the analysis, representative repeats are compared with all cyclic permutations, except for the identity permutation. If the repeat is unique (non-composite), the number of differences between repeats should be similar, regardless of permutations (as shown [file pone.0179173.s001.tif]
