# Supplementary material for: A TALE-inspired computational screen for proteins that contain approximate tandem repeats
Source: PLoS One. 2017 Jun 15;12(6):e0179173. doi: 10.1371/journal.pone.0179173 (PMC5472282; doi:10.1371/journal.pone.0179173)
Supplement: S1 Table — The table presents transactivation domains identified in protein sequences with Nine Amino Acids Transactivation Domain 9aaTAD Prediction Tool http://www.med.muni.cz/9aaTAD. Stringency patterns re described as: Most stringent: MDENQSTYG] {KRHCGP} [ILVFWM] {KRHCGP}{CGP}{KRHCGP}[ILVFWM][ILVFWMAY]{KRHC}, Moderate: [MDENQSTYG] {KRHCGP} [ILVFWM] {KRHCGP} {CGP} {CGP} [ILVFWM] {CGP} {CGP}, Less stringent: [MDENQSTYCPGA] X[ILVFWMAY] {KRHCGP} {CGP} {CGP} [ILVFWMAY]XX. (DOCX) [file pone.0179173.s005.docx]

## S1 Table. TAD prediction.

The table presents transactivation domains identified in protein sequences with Nine Amino Acids Transactivation Domain 9aaTAD Prediction Tool <http://www.med.muni.cz/9aaTAD>. Stringency patterns are described as:

Most stringent: [MDENQSTYG]{KRHCGP}[ILVFWM]{KRHCGP}{CGP}{KRHCGP}[ILVFWM][ILVFWMAY]{KRHC}

Moderate: [MDENQSTYG]{KRHCGP}[ILVFWM]{KRHCGP}{CGP}{CGP}[ILVFWM]{CGP}{CGP}

Less stringent: [MDENQSTYCPGA]X[ILVFWMAY]{KRHCGP}{CGP}{CGP}[ILVFWMAY]XX

| **Seq #** | **Sequence ID** | **Identified TADs** | **Residue numbers** | **Pattern stringency** |
| --- | --- | --- | --- | --- |
| 32 | W1I7I9 | DNLEILATP  THLYEALSY  MAAETILSE | 93-101  159-167  641-649 | Less stringent  Less stringent  Less stringent |
| 37 | G5AAP8 | ESVMEAFPR | 75-83 | Less stringent |
| 38 | C9ZJS6  C9ZJS7  Q586F1  Q586F2 | MLLDGEVWE  MLLDGEVWE  MLLDGEVWE  MLLDGEVWE | 259-267  1116-1124  409-417  237-245 | Most stringent  Most stringent  Most stringent  Most stringent |
| TALE | AEQ98589.1 | QALESIVAQ | 1063-1071 | Most stringent |
| RipTAL | Q8XYE3 | NAMEFLLTA  EFLLTALEF  DWLLQILET | 23-31  26-34  1284-1292 | Moderate  Moderate  Moderate |
| BurrH | E5AW45 | TVLDLELAF | 516-524 | Most stringent |
| MOrTL1 | ECG96325.1 | ENWDALIDL  QAIATLLAK  TTLLEKWAA | 75-83  134-142  269-277 | Moderate  Moderate  Moderate |
| MOrTL2 | EBN91409 | GIFNDLITI  D IFNDLIT | 151-159  150-158 | Moderate  Moderate |
