# Supplementary material for: A TALE-inspired computational screen for proteins that contain approximate tandem repeats
Source: PLoS One. 2017 Jun 15;12(6):e0179173. doi: 10.1371/journal.pone.0179173 (PMC5472282; doi:10.1371/journal.pone.0179173)
Supplement: S1 Text — (DOCX) [file pone.0179173.s006.docx]

## S1 Text. STRING analysis of protein interactions

We carried out STRING analysis [31] of the proteins that contained tandem repeats which passed all the filters (Shannon score, secondary structure prediction, exclusion of known repeat types). We wanted to see whether they associate with proteins that could take part in transport or nuclear processes.

STRING analysis for TALEs typically finds proteins that are transmembrane, receptors, signal peptides, or transporter proteins (ATP-binding). For example, TAL effector B2SU53 interacts with colicin I receptor (transport, membrane, receptor activity), hemagglutinin (protein secretion), transposase, GtrA family protein (transport, membrane component), and trytophan repressor binding protein.

We found no interactors for repeat #37 containing fungal protein G5AAP8. Also, using default stringency settings, we did not find interactors for repeat #38 containing *Trypanosoma* sequences: C9ZJS6, C9ZJS7, Q586F1, Q586F2. Under low stringency settings however, STRING found one interactor, an acyl transferase-like protein (550 aa) EAN77116 (by text mining). For sequence W1I7I9, containing repeat #32, we did not find any interactors as *C. parasitica* is not listed in the STRING database, but STRING search in *Leotimyceta* with W1I7I9 protein sequence identified a half shorter (671 aa) protein (with e value of 0.0) in *Botryotinia fuckeliana*, yet it had no interactors. Another similar protein, a 360 aa protein (e value of 1e-105) from *Sclerotinia sclerotiorum* interacted with low stringency only with a few undescribed hyphotetical proteins.
